# Revisiting the relationship between impulsivity, apathy, and action control: Bayesian inference from a stop-signal task study

**DOI:** 10.1371/journal.pone.0352351

**Published:** 2026-06-23

**Authors:** Emma Michel, Axel Garnier-Allain, Mariia Kaliuzhna, Djamila Bennabi, Mathieu Servant, Matthieu Béreau

**Affiliations:** Laboratoire de Recherches Intégratives en Neurosciences et Psychologie Cognitive, Institut National de la Santé et de la Recherche Médicale, Université Marie et Louis Pasteur, Besançon, France; Nanjing University, CHINA

## Abstract

Apathy and impulsivity are multidimensional constructs that shape goal-directed behavior. Although the motivational dopaminergic spectrum hypothesis frames them as opposing ends of a single continuum, questionnaire-based studies consistently show that they positively co-occur in both healthy and clinical populations. An emerging theoretical resolution of this paradox is that apathy and impulsivity relate to distinct components of goal-directed behavior, with co-occurrence potentially arising from domain-general factors and/or partially overlapping neurophysiological networks. Here, we examined this proposal in the domain of action control using the stop-signal task, which provides separable indices of action inhibition (stop-signal response time, SSRT) and action initiation (go response time, goRT) within the independent race model. Based on prior findings and theoretical considerations, we expected SSRT to covary with urgency-related impulsivity and goRT with action-initiation apathy. A large sample of healthy young adults (N = 144) completed a stop-signal task implemented in accordance with consensus recommendations, alongside validated multidimensional measures of impulsivity (UPPS-P impulsive behavior scale) and apathy (Lille Apathy Rating Scale). Bayesian analyses replicated a positive association between global apathy and impulsivity scores. However, SSRT was not associated with urgency, and goRT was not associated with action-initiation apathy, with moderate evidence for the null. Additional exploratory analyses provided moderate evidence against the majority of correlations between stop-signal indices and the remaining scale dimensions, including global scores. Thus, stop-signal indices did not dissociate apathy and impulsivity in action control, highlighting limits of task-based accounts of their co-occurrence.

## Introduction

Impulsivity and apathy are two multifaceted psychological constructs that play a critical role in shaping decision-making, behavioral regulation, and goal-directed actions. Impulsivity is broadly characterized as a tendency to engage in rapid, unplanned responses to internal or external stimuli, often without sufficient consideration of potential negative consequences (International Society for Research on Impulsivity, 2019). In contrast, apathy is marked by diminished motivation, lack of initiative, and emotional indifference, leading to reduced engagement in daily life and social activities [1–4]. Both constructs can be studied in healthy individuals and in clinical populations. They have received considerable attention because they play an important role in goal-directed behavior and are associated with functional outcomes in various neurological and psychiatric disorders [5–8].

Traditionally, impulsivity and apathy have been regarded as opposing ends of a motivational spectrum, primarily mediated by the dopaminergic system. Apathy is associated with a hypodopaminergic state, while impulsivity is associated with a hyperdopaminergic state [9,10]. This assumption, referred to as the motivational dopaminergic spectrum hypothesis, has been challenged by recent data. Petitet et al. [7] conducted a large-scale study in the general population and found a positive correlation between global measures of apathy and impulsivity, as assessed by commonly used self-report questionnaires. A follow-up analysis showed that this positive association remained significant for most sub-domains. Similar results were obtained using smaller samples [8,11]. These results challenge the motivational dopaminergic spectrum hypothesis, and highlight the need for more research on the relationship between apathy and impulsivity. This research is important because the co-occurrence of these two psychological constructs has also been observed in several neuropsychiatric disorders including Parkinson’s disease, Alzheimer’s disease, Huntington disease, as well as frontotemporal lobar degeneration, and substantially impact functional outcomes [12–18]. A precise understanding of the neurocognitive mechanisms underlying this co-occurrence is required to develop efficient diagnostic and therapeutic strategies.

One way to study the relationship between impulsivity and apathy at the mechanistic level is to use an experimental task that allows for the expression of both traits. To our knowledge, only one study has employed this approach. Petitet et al. (2012) used an effort-based variant of the Traffic Light Task, in which (healthy) subjects had to squeeze a hand-held dynamometer when the color of a traffic light changed to green. A premature response (before green onset) was penalized by a small monetary loss. Valid responses were rewarded based on an exponential decay function, with the reward and the decay rate being manipulated at different timescales. The results revealed a negative correlation between impulsivity scores and response times (RTs), indicating that higher impulsivity levels were associated with shorter RTs. This association was not modulated by experimental conditions, and follow-up analyses showed that the faster RTs for impulsive individuals were not due to faster sensorimotor processing, but instead to a tendency to anticipate the green light. By contrast, higher apathy scores were associated with a reduced modulation of response vigor by reward magnitude. The independent signatures of impulsivity and apathy on task performance suggest that each maps onto a distinct component of goal-directed behavior. Although these components can vary independently, domain-general influences (e.g., chronic stress or available attentional resources) and/or partially shared neurophysiological substrates may underlie their co-expression [8,13,17], thereby helping to explain the positive correlation between self-report measures.

**Fig 1:**
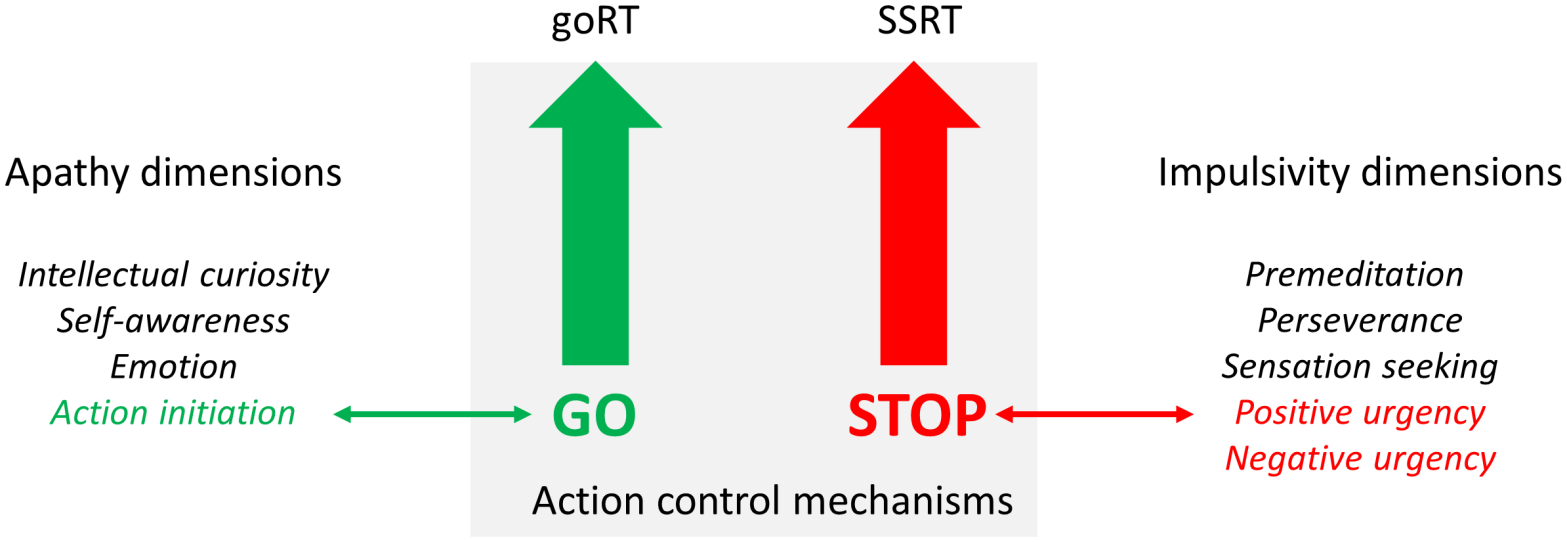
Expected relationship between impulsivity and apathy constructs and action control mechanisms. The central component of the figure depicts the two core processes of the independent race model for the stop-signal task. The GO process leads to action initiation, while the STOP process leads to action inhibition. The response time in GO trials, referred to as goRT, is a proxy for the efficiency of the GO process, while the stop signal response time (SSRT) is a proxy for the efficiency of the STOP process. The peripheral components of the figure describe the dimensions of apathy and impulsivity, as measured via the LARS and the UPPS-P scales, respectively. We hypothesize an association between mean goRT and the action initiation dimension of the LARS, and an association between SSRT and the negative–positive urgency dimensions of the UPPS.

The present work expands upon this methodological approach by exploring another experimental task that may allow for the simultaneous expression of impulsivity and apathy in healthy individuals through different cognitive mechanisms that support goal-directed behavior. The stop-signal task was originally developed to study the ability to stop unwanted actions, a process known as response inhibition [19,20]. In this task, subjects perform a standard 2-choice RT task (e.g., press the left key if the arrow presented at the center of the screen points to the left, press the right key if the arrow points to the right). On a small proportion of trials (typically 25%), the arrow stimulus is replaced by a stop signal (e.g., a change in stimulus color or shape) after a variable stop-signal delay, prompting subjects to withhold their response. The time required to internally stop the formation of the motor plan, referred to as stop-signal RT (SSRT), serves as an index of *action inhibition efficiency*. A longer SSRT indicates reduced inhibitory efficiency. This latency cannot be observed, and must be inferred using a computational model of information processing in the task. The independent race model has been developed for this purpose [20]. The model assumes two independent processes, a go and a stop process, that race against each other (Fig 1). If the stop process finishes before the go, the response is inhibited (successful stop trial). If the go process finishes before the stop, the response is given (unsuccessful stop trial). For each subject, the model provides an estimate of the SSRT. Importantly for present purposes, the stop-signal task also offers an independent measure of the *speed of action initiation*, which corresponds to the mean latency of responses in go trials (denoted mean goRT). By leveraging empirical evidence and theoretical frameworks from the fields of cognitive and personality psychology, we can relate measures of impulsivity and apathy to SSRT and mean goRT respectively. The rationale underlying these associations is detailed below.

Logan et al. [21] hypothesized that impulsivity was caused by a decreased ability to inhibit actions, rather than by an improved ability to execute prepotent responses. In support of this hypothesis, they reported a significant positive correlation (*r* = .315) between impulsivity scores and SSRT in a large sample of university students (*N* = 136), but no significant association between impulsivity scores and mean goRT (*r* = −.036). While some subsequent studies replicated these findings [22–26], others failed to find a significant correlation between impulsivity scores and SSRT [27–30]. There are at least four explanations for these discrepant findings. First, although all of the above-cited studies focused on healthy adult participants, there are considerable variations in sample size between them. While the original study by Logan et al. (2017) used a sample of 136 students, all subsequent studies except one [30] relied on smaller samples (*N* = 20–92), raising the possibility that some null findings reflect insufficient statistical power rather than a true absence of effects. Second, some studies selected participants from large pools based on extremely low or high impulsivity scores [27–29], while others did not [21–24,25,26,30]. Third, the design and analysis of the stop-signal task varies considerably between studies, and some of these variations are known to affect the validity of the SSRT estimates [31]. Finally, different questionnaires have been used to estimate impulsivity, and it is unclear how these measures relate to each other. Some studies have focused on global impulsivity scores, while others have leveraged the multidimensional nature of impulsivity to examine how SSRT relates to specific facets of action control. This multidimensional nature is supported by neuroscientific, clinical, and psychometric findings [5,32, but see 33]. For instance, Whiteside and Lynam [34] administered several widely used impulsivity questionnaires to a large student sample and performed a factor analysis on the data. This analysis identified a four-factor structure underlying impulsivity-like behavior: negative Urgency (tendency to act rashly when experiencing intense negative emotions), lack of Premeditation (diminished tendency to pause and engage in careful thought or planning before taking action), lack of Perseverance (reduced ability to stick with a task until it is finished), and Sensation seeking (tendency to seek adventurous and exciting experiences). Higher scores on each dimension indicate greater impulsivity. The resulting UPPS scale has been applied in combination with the stop-signal task. Wilbertz et al. [29] reported a significant positive correlation between mean SSRT and the negative urgency dimension, highlighting the importance of considering the different impulsivity facets. A subsequent study showed that this effect intensifies when subjects are placed in threatening conditions [35]. However, Wüllhorst et al. [30] failed to find significant relationships between SSRT and any of the UPPS dimensions. It should be noted that the null findings reported thus far are based on *p*-values exceeding the  .05 threshold. However, non-significant *p*-values do not quantify the evidence in support of the null hypothesis; they simply indicate that the data do not *p*rovide sufficient evidence to reject the null.

The inconsistent findings regarding the relationship between impulsivity and SSRT highlight the need for further research. In the present work, we addressed the aforementioned methodological limitations in four ways. First, our stop-signal task design and analysis methods strictly adhered to recent recommendations from a consensus guide [31]. Second, we modeled our sample size and characteristics after those in the seminal study by Logan et al. (2017) that reported positive results. Third, we performed Bayesian analyses of the data to quantify the evidence for or against each effect of interest. Finally, the scale used to measure the different facets of impulsivity, the UPPS-P, reflects theoretical advances over the original UPPS framework (Lynam et al. [Unpublished]), and a short-version has been validated for use in our target (French) population [36]. The UPPS-P adds a fifth factor, Positive urgency, which reflects the tendency to act impulsively under intense positive emotions, although positive and negative urgency are substantially correlated. A higher score on each subscale indicates a greater level of the corresponding impulsivity-related dimension. Based on our literature review, we predicted a positive correlation between SSRT and impulsivity, which should be most apparent when focusing on the urgency dimension.

Another distinctive feature of our study is its concurrent examination of apathy. While impulsivity is typically found to be unrelated to mean goRT, there are theoretical reasons to predict an association between this latency and apathy. Similar to impulsivity, factor-analytic work on apathy questionnaires supports a multidimensional structure, motivating dimension-specific tests. We assessed apathy using the Lille Apathy Rating Scale (LARS), a semi-structured interview originally developed in a clinical context and validated in a sample including patients with Parkinson’s disease and healthy control participants [37]. We selected this instrument because, when the study was designed, it was a multidimensional apathy measure available in French and appropriate for the aims of the present study. In addition, using an interviewer-administered apathy measure and a self-report impulsivity measure reduced the risk that any apathy–impulsivity association would be driven solely by shared self-report method variance. The LARS comprises four dimensions: lack of intellectual curiosity (low interest in novelty and a reduced drive for acquiring knowledge), reduced self-awareness, reduced emotional responses, and reduced action initiation (low everyday productivity, lack of initiative). Higher scores on each subscale indicate greater severity along the corresponding apathy dimension. Because action-initiation apathy appears to share processing demands with the go process in the independent race model of the stop-signal task, we predicted a positive correlation between scores on this subscale and mean goRT. Thus, we tested whether impulsivity and apathy relate to dissociable components of action control (Fig 1).

## Materials and methods

### Participants

One hundred and forty-four undergraduate psychology students from Marie and Louis Pasteur University were recruited between 1 June 2022 and 28 April 2024 and took part in the study in exchange for course credit (128 females; mean age = 18.7 years, SD = 2.04, range = 18−37 years). Inclusion criteria were age between 18 and 40 years, normal or corrected-to-normal vision, and no history of neuropsychiatric disorders. Twelve subjects were excluded after data collection because their performance on the stop-signal task did not meet pre-specified criteria intended to increase the validity of SSRT estimates. These exclusion criteria were defined prior to data collection and were based on recommendations from Verbruggen et al. [31]. Specifically, participants were excluded if any of the following occurred: (i) their mean RT on unsuccessful stop trials was numerically longer than their mean goRT (violating the race model independence assumption), (ii) their probability of responding on stop trials fell outside the  .25–.75 range, or (iii) their proportion of go omissions (i.e., go trials with no response within the RT deadline) exceeded 10%. The study was approved by the university ethics committee for research (Agreement No. CERUBFC-2022-01-18-002). All participants provided written informed consent prior to participation.

### Questionnaire measures of apathy and impulsivity

Apathy and impulsivity were assessed using two French-validated scales. Impulsivity was measured using a short French version of the UPPS-P scale [36]. This short version comprises a 20-item self-report questionnaire scored on a 4-point Likert scale (total scores ranging from 20 to 80), subdivided into five dimensions: negative urgency, (lack of) premeditation, (lack of) perseverance, sensation seeking, and positive urgency. Apathy was measured using the LARS scale [37], a semi-structured interview originally validated in French in Parkinson’s disease, with healthy control participants also included in the initial study. The LARS later served as the basis for the Apathy Motivation Index (AMI; Ang et al., 2017), a self-report measure developed for use in the general population and subsequently validated in French [38]. Recruitment for the present study began on 1 June 2022, before publication of the French validation of the AMI, and we therefore retained the LARS for the present study. The LARS comprises 33 questions grouped into nine subscales: everyday productivity, interest, initiative, novelty seeking, efforts and motivation, emotional responses, concern, social life, and self-awareness. Responses are scored using standardized anchored response options, yielding a global apathy score (range: −36–36). Composite factor scores can be derived to index four apathy-related dimensions: intellectual curiosity, self-awareness, emotional responsiveness, and action initiation. Whereas the UPPS-P primarily indexes relatively stable impulsivity-related dispositions, the LARS refers to thoughts, emotions, and activities over the previous four weeks. Accordingly, the present analyses relate impulsivity traits to recent apathy-related variation rather than two perfectly matched trait measures.

### Apparatus and procedure

Testing took place in a quiet room with only the participant and the experimenter present. The UPPS-P was self-administered using a paper-and-pencil format, whereas the LARS was administered by the experimenter. The stop-signal task was presented on a 34.42 × 19.36 cm LCD monitor (resolution: 1920 × 1080 pixels, refresh rate: 60 Hz), at an approximate viewing distance of 60 cm, using an open-source software developed by Verbruggen et al. [31].

### Stop-signal task

**Fig 2:**
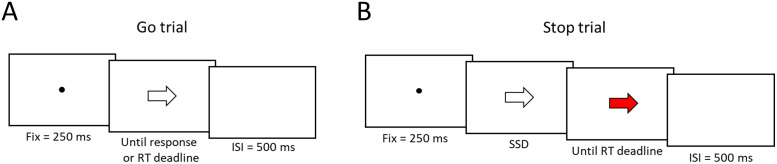
Time course of go and stop trials in the stop-signal task. **A)** Go trials. Each trial began with a central fixation point (Fix) presented for 250 ms, followed by the arrow stimulus. Participants had a maximum of 1250 ms (RT deadline) to respond. The inter-stimulus interval (ISI) was set to 500 ms. **B)** Stop trials. The sequence was identical to go trials, except that the arrow stimulus turned red after a variable stop-signal delay (SSD), signaling participants to withhold their response. This stop signal remained on the screen until the RT deadline.

Participants completed a stop-signal task consisting of a training phase followed by experimental blocks. The task design and procedure followed current recommendations [31]. The training phase included 32 trials (24 go trials, 8 stop trials) and provided immediate feedback (750 ms) after each response (“Correct response,” “Incorrect response,” “Response too slow,” “Response too fast,” “Well done, response stopped,” or “Careful, you must stop your response”), allowing participants to familiarize themselves with the task demands and timing constraints. The experimental phase comprised four blocks of 64 trials each (48 go trials and 16 stop trials), separated by 15-s breaks. Trials were presented in a randomized order. Between blocks, participants received feedback on their mean RT (in milliseconds), the proportion of omission trials (with a reminder that this value should be 0), and the proportion of successfully inhibited responses following the stop signal (with a reminder that this value should be close to  .5). Each trial began with the presentation of a fixation point (0.6 cm in diameter) at the center of a white background for 250 ms. This was followed by the appearance of a white arrow with black edges (4.6 cm in length and 3.2 cm in height) at the center of the screen. On go trials, the arrow remained on the screen until the participant responded, with a maximum presentation time of 1250 ms (RT deadline; Fig 2A). Participants were instructed to press the left arrow key on a standard French keyboard with their right index finger when the stimulus pointed left, and the right arrow key with their right middle finger when the stimulus pointed right. They were explicitly instructed not to wait for a potential stop signal, but instead to respond as quickly and accurately as possible to the direction of each arrow. On stop trials, the white arrow turned red (RGB: 255,38,0) after a variable stop-signal delay (SSD), prompting participants to inhibit their response (Fig 2B). The SSD was initially set to 300 ms, and was continuously adjusted using a staircase tracking procedure to reduce predictability of stop trials and to maintain a stopping success rate close to 50%. Specifically, the SSD increased by 50 ms following successful inhibition and decreased by 50 ms following failed inhibition. The stop signal remained on the screen until the RT deadline. The inter-trial interval was fixed at 500 ms. Overall, the task included 192 go trials and 64 stop trials. The SSRT was estimated using the integration method, with go omissions replaced as recommended. The mean goRT included both correct and incorrect trials to the choice task. Statistical conclusions were unchanged when mean goRT was computed using correct trials only.

### Statistical analyses

Statistical analyses were conducted using JASP v.0.19.1. Data were initially analyzed using Bayesian linear regression models to control for age and sex. However, because (i) these analyses may be difficult to interpret for readers without a background in Bayesian statistics and (ii) age and sex had a negligible impact on the results, we report the simpler Bayesian correlation analyses instead. We opted for Bayesian Kendall’s τ-b correlation analyses since we did not assume linear relationships between variables and were primarily interested in monotonic associations. For the Bayesian Kendall’s τ-b correlation analyses, we used the default stretched beta prior implemented in JASP (width = 1; [39]). In addition, Kendall’s τ-b is well suited for the analysis of global impulsivity–apathy scores and their subdimensions, which are derived from ordinal-scale questionnaire data and may include tied ranks.

## Results

### Sample characteristics

We planned to analyze data from 136 undergraduate psychology students, similar to Logan et al. (2017). To account for expected exclusions based on the criteria described by [31]; see Methods), which are intended to improve the validity of stop-signal task analyses, we increased the sample size to 144, anticipating an exclusion rate of approximately 6%. The actual rejection rate was slightly higher, leading to a final sample of 132 participants. Demographic characteristics and descriptive statistics for the final sample, including UPPS-P and LARS total and dimension scores, are presented in Table 1.

**Table 1:** Demographic characteristics and descriptive statistics for the final sample (N = 132).

Variable	Mean (SD) or n (%)	Range
Women	113 (85.6%)	—
Men	19 (14.4%)	—
Age, years	18.6 (1.3)	18–25
Years of education	13.3 (1.3)	9–20
UPPS-P total score	46.8 (7.7)	28–69
Positive urgency	11.1 (2.5)	4–16
Negative urgency	9.6 (3.2)	3–16
Lack of perseverance	7.6 (2.3)	4–16
Lack of premeditation	7.8 (2.5)	4–16
Sensation seeking	10.8 (2.5)	5–16
LARS total score	−23.0 (5.5)	−34 to −5
Intellectual curiosity	−2.3 (0.9)	−4–0
Self-awareness	−2.6 (1.4)	−4–2
Emotional responsiveness	−3.0 (1.1)	−4–1
Action initiation	−2.6 (1.0)	−4 to 0.5

### Global measures of apathy and impulsivity are positively associated

**Fig 3:**
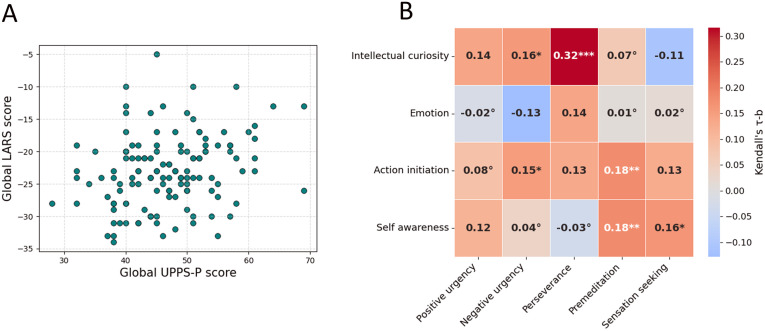
Relationship between global and dimension-level scores of the UPPS-P and LARS scales. **(A)** Scatter plot of global LARS and UPPS-P scores. Each point represents one participant. **(B)** Heatmap illustrating Kendall’s τ-b correlations between UPPS-P and LARS dimensions. Each cell shows the correlation coefficient (rounded to two decimals). Asterisks indicate increasing strength of evidence for the presence of a correlation (BF_10_ > 3: *; > 10: **; > 30: ***), whereas degree symbols indicate evidence favoring the absence of a correlation (BF_10_ < 1/3: °; < 1/10: °°; < 1/30: °°°).

We first conducted a Bayesian Kendall’s τ-b correlation between global LARS and UPPS-P scores. This analysis returns a Bayes Factor, noted BF_10_, quantifying the evidence for the presence of a correlation (H_1_) relative to the null hypothesis of no correlation (H_0_). Following standard conventions, we interpreted BF_10_ values of 1–3 as anecdotal evidence, 3–10 as moderate evidence, 10–30 as strong evidence, and > 30 as very strong evidence in favor of H_1_. Conversely, values < 1 were interpreted as evidence in favor of H0, with the same cutoffs applying to BF_01_ (i.e., the reciprocal of BF_10_). The analysis also returns a point estimate of the Kendall’s *τ*-b coefficient, as well as a measure of uncertainty in the form of a 95% credible interval (CI). Consistent with previous work [7,8], we found a positive correlation between global LARS and UPPS-P scores, (*τ*-b = .186, 95% CI =  [.068,  .295]; Fig 3A), and the evidence for this effect was strong (BF_10_ = 16.02).

### Most LARS and UPPS-P dimensions positively correlate

We next evaluated the Kendall’s *τ*-b correlation matrix between the LARS and UPPS-P dimensions. Of the 20 Kendall’s *τ*-b coefficients, only four were negative (Fig 3B). Among these, the Bayes factor analysis provided anecdotal evidence in favor of two correlations and moderate evidence against the other two. Since anecdotal evidence is generally considered inconclusive, the data did not support any negative association between LARS and UPPS-P dimensions, contrary to the motivational dopaminergic spectrum hypothesis. By contrast, out of the 16 positive Kendall’s *τ*-b coefficients, moderate to strong evidence was found in support of six correlations. The strongest evidence was found for the positive correlation between the LARS intellectual curiosity dimension and the UPPS-P lack of perseverance dimension (*τ*-b = .316, 95% CI =  [.196,  .422], BF_10_ = 1.93 × 10^5^), indicating that individuals with lower curiosity and a diminished drive to acquire new knowledge tend to exhibit reduced persistence in completing tasks. Additionally, there was strong evidence for positive associations between the UPPS-P lack of premeditation dimension and both the LARS action initiation (*τ*-b = .177, 95% CI = [.060,  .287], BF_10_ = 10.32) and self-awareness (*τ*-b = .180, 95% CI = [.063,  .290], BF_10_ = 12.15) dimensions. These suggest that individuals who are less inclined to pause and think before acting also tend to show lower everyday productivity, reduced initiative, and diminished metacognitive insight. Finally, the analysis revealed moderate evidence for positive correlations between the UPPS-P negative urgency dimension and both the LARS action initiation (*τ*-b = .152, 95% CI = [.036,  .262], BF_10_ = 3.19) and intellectual curiosity (*τ*-b = .158, 95% CI = [.042,  .268], BF_10_ = 4.17) dimensions. Taken together, these findings account for the positive correlation observed between the global UPPS-P and LARS scores.

### Stop-signal task performance

The stop-signal task was implemented in accordance with recent consensus recommendations ([31]; see Materials and Methods for full procedural details). Each participant contributed a total of 192 go trials and 64 stop trials. Descriptive stop-signal performance statistics for the final sample are presented in Table 2.

**Table 2:** Descriptive statistics for stop-signal task performance.

Measure	Mean (SD)
p(respond|signal)	0.490 (0.037)
Stop-signal delay, ms	306.3 (150.5)
Stop-signal response time, ms	230.9 (36.3)
RT on unsuccessful stop trials, ms	465.9 (111.2)
Go RT (all go trials with a response), ms	553.6 (140.8)
Go RT (correct go trials only), ms	555.0 (140.0)
Go RT intra-subject SD, ms	135.9 (48.7)
Go omissions, %	0.99 (1.85)
Go errors, %	1.24 (1.80)

p(respond|signal) = probability of responding on a stop trial; RT = reaction time; ms = milliseconds. Go RT intra-subject SD refers to within-participant variability in go-trial response latencies. p(respond|signal) was close to 0.5 on average, consistent with the expected convergence of the staircase procedure.

### SSRT estimates do not correlate with the urgency dimensions from the UPPS-P scale

**Fig 4:**
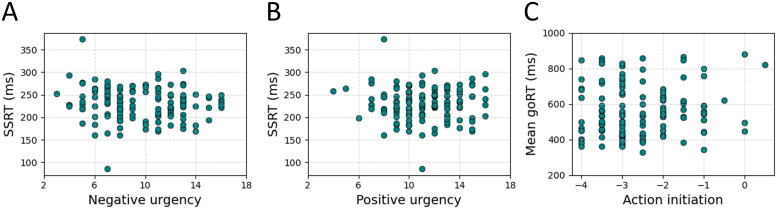
Hypothesis-relevant scatterplots. **(A)** Stop-signal response time (SSRT) as a function of negative urgency. **(B)** SSRT as a function of positive urgency. **(C)** Mean goRT as a function of action initiation. Each point represents one participant. ms = milliseconds.

We hypothesized a positive correlation between SSRT estimates from the stop-signal task and negative–positive urgency dimensions from the UPPS-P. For each subject, the SSRT was estimated using an integration procedure that complies with the recommendations from [31]; see Methods). Bayesian Kendall’s τ-b correlation analyses showed moderate evidence against a correlation between SSRT and the UPPS-P negative urgency dimension (*τ*-b = −.074, 95% CI = [−.186,  .041], BF_10_ = 0.251; Fig 4A), and moderate evidence against a correlation between SSRT and the UPPS-P positive urgency dimension (*τ*-b = .034, 95% CI = [−.080,  .147], BF_10_ = 0.135; Fig 4B). Because negative and positive urgency dimensions correlated substantially (*τ*-b = .295, 95% CI = [.175,  .401], BF_10_ = 29128), in line with previous work [36], we examined the correlation between SSRT and a general urgency measure obtained by averaging across the two dimensions. Moderate evidence for the null hypothesis of no correlation was still obtained (*τ*-b = −.068, 95% CI = [−.234,  .103], BF_10_ = 0.146). Finally, we compared the SSRT of subjects scoring low or high on each urgency measure (based on the 25^th^ and 75^th^ percentiles). Participants in the bottom 25% of scores were assigned to the low urgency group, and those in the top 25% were assigned to the high urgency group. Bayesian Mann-Whitney U tests were inconclusive for each urgency measure (negative urgency: BF_10_ = 1.92; positive urgency: BF_10_ = 2.02; general urgency: BF_10_ = 1.96). Taken together, these analyses indicate that the urgency dimensions of the UPPS-P scale are not associated with action inhibition, as measured by SSRT. Given that the full sample provided only moderate evidence for the absence of an association, and that analyses restricted to participants with low or high urgency scores were inconclusive, these findings suggest that much of the prior research in this field may have been underpowered.

### Go mean RT estimates do not correlate with the action initiation dimension from the LARS scale

We also predicted a positive correlation between mean goRT and the action initiation dimension of the LARS scale. However, the Bayesian Kendall’s τ-b correlation analysis showed moderate evidence against such a correlation (*τ*-b = .084, 95% CI = [−0.032,  .195], BF_10_ = 0.313; Fig 4C). Similar to the previous analysis of urgency, we repeated this analysis focusing on subjects scoring low or high on the action initiation dimension (based on the 25^th^ and 75^th^ percentiles). The Bayesian Mann-Whitney U test was inconclusive (BF_10_ = 0.744).

### Correlations between stop-signal performance and other dimensions of impulsivity and apathy

**Fig 5:**
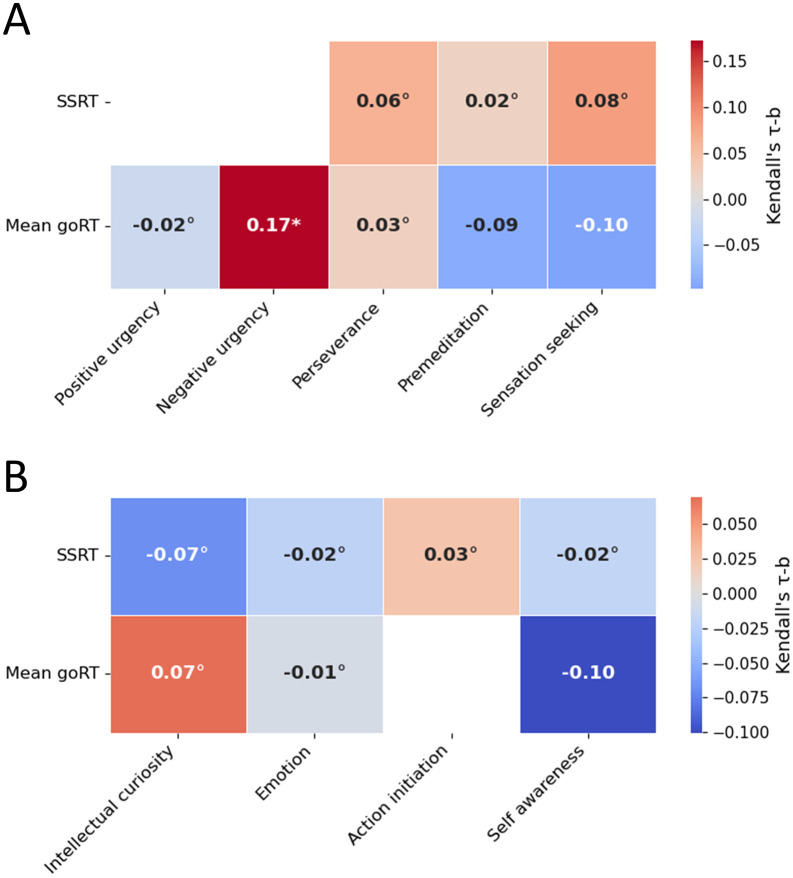
Exploratory correlations between the primary stop-signal task performance measures and other UPPS-P and LARS dimensions. Heatmaps illustrating Kendall’s τ-b correlations between the primary stop-signal task performance measures (stop-signal response time [SSRT] and mean goRT) and the UPPS-P (panel A) and LARS (panel B) dimensions. Each cell shows the correlation coefficient (rounded to two decimals). Asterisks indicate increasing strength of evidence for the presence of a correlation (BF_10_ > 3: *; > 10: **; > 30: ***), whereas degree symbols indicate evidence favoring the absence of a correlation (BF_10_  < 1/3: °; < 1/10: °°; < 1/30: °°°). Correlations that were previously targeted by our hypotheses are excluded.

Finally, in an exploratory analysis, we assessed correlations between the primary stop-signal task performance measures (SSRT and mean goRT) and other dimensions, as well as the global scores, of the LARS and UPPS-P scales. Moderate evidence for the absence of a correlation was consistently obtained between performance measures and global scores (SSRT vs. UPPS-P: *τ*-b = .044, 95% CI = [−0.071,  .156], BF_10_ = 0.149; SSRT vs. LARS: *τ*-b = −.014, 95% CI = [−.128,  .099], BF_10_ = 0.117; mean goRT vs. UPPS-P: *τ*-b = −.031, 95% CI = [−.144,  .083], BF_10_ = 0.131; mean goRT vs. LARS: *τ*-b = .054, 95% CI = [−.061,  .166], BF_10_ = 0.172). Fig 5A shows the Kendall’s *τ*-b correlation matrix between performance measures and UPPS-P dimensions, whereas Fig 5B shows the Kendall’s *τ*-b correlation matrix between performance measures and LARS dimensions. Correlations that were targeted by our hypotheses have been excluded from these plots. The only correlation for which we found (moderate) evidence was a positive association between mean goRT and the negative urgency dimension of the UPPS-P scale (*τ*-b = .173, 95% CI = [0.056,  .282], BF_10_ = 8.24). This effect is somewhat counterintuitive, as it indicates that participants with higher negative urgency, who are generally prone to acting impulsively under intense negative emotions, responded more slowly on average in go trials. To gain further insight into this effect, we examined the correlation between negative urgency and the proportion of incorrect responses in go trials. Moderate evidence was observed for a negative association (*τ*-b = −0.152, 95% CI = [−0.262, −0.036], BF_10_ = 3.14), indicating that participants with higher negative urgency tended to make fewer errors. This finding suggests a speed-accuracy tradeoff: individuals high in negative urgency slow their responses in go trials, which in turn leads to improved accuracy. Among the 14 remaining correlations, moderate evidence for the null hypothesis was found in 11, with the rest being inconclusive. Given the exploratory nature of these analyses and the number of tests conducted, the positive association between mean goRT and negative urgency should be interpreted as hypothesis-generating and requiring independent replication.

**Fig 6:**
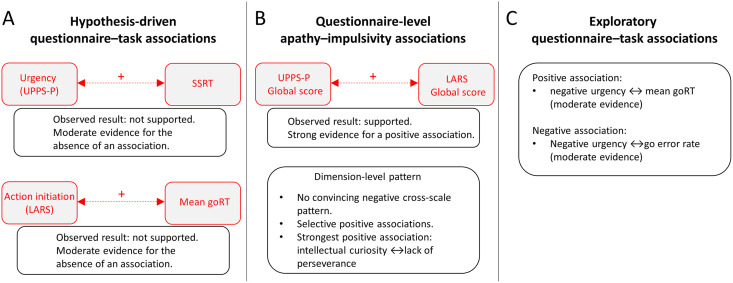
Integrated schematic overview of the key findings. Red dashed arrows indicate associations that were expected based on our hypotheses. The plus signs indicate the predicted positive direction of these associations. **(A)** Summary of the two hypothesized questionnaire–task associations: urgency with stop-signal response time (SSRT), and action-initiation apathy with mean goRT. Neither was supported. **(B)** Summary of the questionnaire-level apathy–impulsivity associations: global UPPS-P and LARS scores were positively associated, and the dimension-level pattern showed selective positive associations without a convincing negative cross-scale pattern. **(C)** Summary of the main exploratory questionnaire–task associations: negative urgency was positively associated with mean goRT and negatively associated with the proportion of go errors.

To facilitate interpretation of the overall pattern of results and ease the transition to the Discussion, Fig 6 provides an integrated schematic overview of the key hypothesis-driven and exploratory findings presented above.

## Discussion

The present study tested whether specific dimensions of impulsivity and apathy relate to dissociable components of action control within the independent race model framework [20]. We predicted that SSRT would correlate positively with the negative/positive urgency facets of impulsivity and that mean goRT would correlate positively with action-initiation apathy. Bayesian correlation analyses yielded two main findings, which are discussed below.

### Questionnaire dimensions do not map onto stop-signal indices

The first, and most directly relevant to our mechanistic hypotheses, is that the predicted mappings between questionnaire dimensions and stop-signal indices were not supported. Specifically, SSRT did not show the anticipated positive association with either negative urgency or positive urgency, and evidence was instead moderate in favor of the null hypothesis of no association. Likewise, mean goRT did not show the predicted positive association with the action-initiation dimension of apathy, with moderate evidence again favoring the absence of a relationship. These results suggest that, in healthy young adults, urgency-related impulsivity and action-initiation apathy are not straightforwardly reflected in SSRT or mean goRT under neutral task conditions.

This null pattern is informative in light of the mixed literature linking impulsivity to SSRT. The original claim that impulsivity primarily reflects a reduced ability to inhibit actions was supported by a positive association between global impulsivity and SSRT in Logan et al. [21], and has been replicated in some subsequent studies (e.g., [22,24,25]. However, other studies have failed to detect reliable SSRT–impulsivity relationships [27–29], including work that focused specifically on UPPS-derived facets [30]. The present study addressed several methodological limitations highlighted in this field by adhering closely to the consensus recommendations for stop-signal task design and SSRT estimation [31] and by using Bayesian inference to quantify evidence for both the presence and absence of effects. The current findings refine the state of evidence by indicating that any monotonic association between urgency and SSRT in healthy young adults, if it exists, is unlikely to be reliably expressed in a neutral stop-signal task and may instead depend on affective context, such as when response inhibition is assessed under experimentally induced threat [35]. This interpretation follows from the definition of urgency as the tendency to act rashly under intense emotional states, a process that is minimally engaged in a standard stop-signal task lacking emotional or motivational manipulation. That said, the absence of an urgency–SSRT association under neutral conditions does not imply that urgency-related traits are behaviorally inert in this context. In the present dataset, negative urgency was associated with longer mean goRT and fewer incorrect go responses, consistent with a speed–accuracy trade-off in which individuals higher in negative urgency adopt a more cautious response strategy. Because this association emerged from exploratory analyses involving multiple comparisons rather than from our pre-specified hypotheses, it should be treated as hypothesis-generating pending independent replication. If replicated, it would suggest that, in neutral contexts, urgency-related impulsivity may be expressed as strategic response adjustment, potentially reflecting compensatory caution driven by awareness of everyday impulse-control difficulties, rather than as changes in the latency of the stopping process itself.

A comparable task–construct mismatch, though for different reasons, may explain the apathy findings. The hypothesis that action-initiation apathy would correlate positively with mean goRT was grounded in the idea that action initiation shares processing demands with the GO process in the independent race model. However, goRT in the stop-signal task indexes the speed of responding to external cues, whereas the LARS action-initiation dimension is rather intended to capture reduced spontaneou*s* initiation, productivity, and initiative in daily life. The absence of the predicted association therefore suggests an important dissociation between self-initiated goal pursuit in naturalistic contexts and stimulus-driven response execution in a structured task. This distinction aligns with neurocognitive frameworks in which apathy reflects reduced self-generation of goal-directed behavior despite relatively preserved responding when behavior is externally prompted [2,4,6,40,41].

Additional exploratory analyses provided moderate evidence against the majority of correlations between stop-signal indices and the remaining questionnaire dimensions, including global scores. Consequently, stop-signal indices were largely independent of questionnaire-derived measures in this dataset. This pattern is consistent with accumulating evidence for a dissociation between questionnaire and experimental task measures across various domains [33,42]. Two complementary explanations have been proposed. First, laboratory task performance may be more sensitive to transient contextual influences and therefore reflect very short-term, momentary behavior. By contrast, even questionnaire measures that capture recent variation rather than stable traits, such as the LARS over the previous four weeks, still aggregate behavior over a much broader temporal window than a single laboratory task session. Second, many cognitive paradigms, including the stop-signal task, were designed to maximize robust between-condition effects at the expense of between-person variability, which reduces test–retest reliability (the reliability paradox; [43]) and correlations with trait measures [42]. In a recent large preregistered study, Huang et al. [33] reported lower test–retest reliability for behavioral measures than for self-report measures of impulsivity, consistent with both accounts. Moreover, they found substantially stronger correlations among self-report impulsivity measures than among behavioral tasks, as well as generally low convergence between the two measurement families. Applied to the present data, these considerations offer a parsimonious account for why SSRT and mean goRT can be well-defined and experimentally sensitive indices, yet still show limited correspondence with questionnaire-derived dimensions in a healthy sample tested under neutral conditions.

### Co-occurrence of apathy-related variation and impulsivity traits

The second main finding concerns the positive association between recent apathy-related variation and impulsivity traits. Global LARS and UPPS-P scores were positively associated, with strong Bayesian evidence, although the effect size was modest (τ-b = .186). Given the different measurement formats used (self-report for the UPPS-P and semi-structured interview for the LARS), this association is unlikely to reflect common-method variance. Rather, it replicates and extends earlier reports showing that apathy and impulsivity tend to co-occur in healthy individuals, rather than forming opposite ends of a single continuum [7,8]. Importantly, this prior evidence was obtained using a range of self-report instruments, including the AMI, which was specifically developed for use in healthy populations [Ang et al., 2017]. This suggests that the co-occurrence observed here is not solely driven by the particular apathy instrument used in the present study.

Dimension-level analyses further clarified the structure of this association. Contrary to predictions derived from a simple dopaminergic spectrum account, which posits antagonistic relationships between apathy and impulsivity, negative cross-scale associations were rare and unsupported by convincing evidence. Instead, several associations were positive and supported by moderate-to-strong Bayesian evidence, although their magnitudes were generally small (τ-b = .10–.18). Only one association clearly stood out, linking reduced intellectual curiosity (LARS) with lack of perseverance (UPPS-P), which reached a moderate effect size (τ-b = .32) and was supported by very strong evidence. Overall, this pattern indicates limited and selective overlap across specific facets, rather than a broad or uniform coupling between apathy and impulsivity dimensions.

From a theoretical perspective, this facet-specific and non-antagonistic pattern aligns with recent neuropsychiatric accounts that move beyond a simple dopaminergic continuum between apathy and impulsivity. In particular, apathy has been conceptualized as a disruption of higher-level processes involved in goal-directed inference and policy selection, rather than as a simple reduction in dopaminergic drive [44]. Relatedly, evidence from Parkinson’s disease highlights a role for noradrenergic dysfunction in apathy, through altered precision of expected outcomes [Hezemans et al., 2022]. Although the present data were obtained in healthy young adults and do not permit neurobiological inference, the observed facet-specific and non-antagonistic pattern is more consistent with such multidimensional accounts than with a unitary dopaminergic spectrum.

From a psychometric perspective, these findings echo the conclusions of Petitet et al. [7], who showed that the relationship between apathy and impulsivity is shaped by their multidimensional structure, with global associations obscuring more selective facet-level relationships. The present results similarly support moving beyond global scores and considering the specific motivational and control processes indexed by individual subdimensions.

### Limitations

Several limitations should be noted. First, the sample was strongly female-skewed (Table 1), which may have reduced variability in impulsivity-related traits and may limit the generalizability of the findings. This concern is relevant because sex differences have been reported for some dimensions of impulsivity, although these differences are not uniform across facets or measures [45]. Although sex was included as a covariate in our preliminary regression analyses and had negligible impact on the main results, the marked imbalance in the sample means that the present study was not well suited to evaluating possible sex-specific effects or interactions. Second, the stop-signal task was administered under neutral, non-rewarded conditions. Because both impulsivity and apathy are closely linked to motivational and reward-related processes, the absence of explicit reward contingencies may have reduced the likelihood of observing questionnaire–task associations. This issue may be especially relevant for apathy-related variation, which has been linked to diminished reward sensitivity and reduced invigoration of behavior in rewarded task contexts, and for impulsivity dimensions that are more strongly expressed under affectively or motivationally salient conditions. The present null findings should therefore be interpreted in light of the specific motivational structure of the task. Third, the UPPS-P and LARS do not index perfectly matched temporal levels of analysis, as the UPPS-P is relatively trait-like whereas the LARS captures apathy-related variation over the previous four weeks. This asymmetry should be borne in mind when interpreting both questionnaire–task dissociations and cross-questionnaire associations. Finally, because the LARS dimension scores showed limited variability in this healthy sample, even Kendall’s τ-b may have had reduced sensitivity to detect very small monotonic associations.

## Conclusions

In summary, the present study provides Bayesian evidence that impulsivity-related trait dimensions and recent apathy-related variation do not map straightforwardly onto stop-signal task indices in healthy young adults tested under neutral conditions, despite reliable co-occurrence of these constructs when assessed with self-report (UPPS-P) and interview-based (LARS) measures. Together, these findings delineate important boundary conditions on questionnaire–task relationships in action control, highlighting the context sensitivity of urgency-related effects, the dissociation between self-initiated goal-directed behavior and stimulus-driven response execution, and the limited correspondence between experimental task measures and self-reported traits. More broadly, the results support multidimensional accounts of impulsivity and apathy, emphasizing selective facet-level overlap rather than a unitary dopaminergic spectrum.
