# Supplementary material for: Revisiting the relationship between impulsivity, apathy, and action control: Bayesian inference from a stop-signal task study
Source: PLoS One. 2026 Jun 23;21(6):e0352351. doi: 10.1371/journal.pone.0352351 (PMC13289935; doi:10.1371/journal.pone.0352351)
Supplement: Peer review history

# Peer review: Decision Letter 0

**DOI:** 10.1371/journal.pone.0352351.r001

**Authors:** Yansong Li

12 Mar 2026

-->

PONE-D-26-02739

Revisiting the relationship between impulsivity, apathy, and action control: Bayesian inference from a stop-signal task study

PLOS One

Dear Dr. Servant,

Thank you for submitting your manuscript to PLOS ONE. After careful consideration, we feel that it has merit but does not fully meet PLOS ONE’s publication criteria as it currently stands. Therefore, we invite you to submit a revised version of the manuscript that addresses the points raised during the review process.

Please submit your revised manuscript by Apr 25 2026 11:59PM. If you will need more time than this to complete your revisions, please reply to this message or contact the journal office at plosone@plos.org. When you’re ready to submit your revision, log on to https://www.editorialmanager.com/pone/ and select the 'Submissions Needing Revision' folder to locate your manuscript file.

Please include the following items when submitting your revised manuscript:

- A letter that responds to each point raised by the academic editor and reviewer(s). You should upload this letter as a separate file labeled 'Response to Reviewers'.
- A marked-up copy of your manuscript that highlights changes made to the original version. You should upload this as a separate file labeled 'Revised Manuscript with Track Changes'.
- An unmarked version of your revised paper without tracked changes. You should upload this as a separate file labeled 'Manuscript'.

If you would like to make changes to your financial disclosure, please include your updated statement in your cover letter. Guidelines for resubmitting your figure files are available below the reviewer comments at the end of this letter.

If applicable, we recommend that you deposit your laboratory protocols in protocols.io to enhance the reproducibility of your results. Protocols.io assigns your protocol its own identifier (DOI) so that it can be cited independently in the future. For instructions see: https://journals.plos.org/plosone/s/submission-guidelines#loc-laboratory-protocols. Additionally, PLOS ONE offers an option for publishing peer-reviewed Lab Protocol articles, which describe protocols hosted on protocols.io. Read more information on sharing protocols at https://plos.org/protocols?utm_medium=editorial-email&utm_source=authorletters&utm_campaign=protocols.

We look forward to receiving your revised manuscript.

Kind regards,

Yansong Li

Academic Editor

PLOS One

**Journal Requirements:**

When submitting your revision, we need you to address these additional requirements.

1. Please ensure that your manuscript meets PLOS ONE's style requirements, including those for file naming. The PLOS ONE style templates can be found at

https://journals.plos.org/plosone/s/file?id=wjVg/PLOSOne_formatting_sample_main_body.pdf and

https://journals.plos.org/plosone/s/file?id=ba62/PLOSOne_formatting_sample_title_authors_affiliations.pdf

2. Thank you for stating the following financial disclosure:

“This work was funded by the HORIZON EUROPE European Research Council (ERC Starting Grant 101039226 allocated to Mathieu Servant).”

Please state what role the funders took in the study.  If the funders had no role, please state: 'The funders had no role in study design, data collection and analysis, decision to publish, or preparation of the manuscript.'

If this statement is not correct you must amend it as needed.

Please include this amended Role of Funder statement in your cover letter; we will change the online submission form on your behalf.

3. We note that you have referenced (Lynam, D. R., Smith, G. T., Whiteside, S. P., & Cyders, M. A. (2006). The UPPS-P: Assessing Five Personality Pathways to Impulsive Behavior [Technical report or Unpublished manual]. Purdue University) which has currently not yet been accepted for publication. Please remove this from your References and amend this to state in the body of your manuscript: (ie “Bewick et al. [Unpublished]”) as detailed online in our guide for authors

http://journals.plos.org/plosone/s/submission-guidelines#loc-reference-style

4. Please ensure that you refer to Figure 4 in your text as, if accepted, production will need this reference to link the reader to the figure.

5. If the reviewer comments include a recommendation to cite specific previously published works, please review and evaluate these publications to determine whether they are relevant and should be cited. There is no requirement to cite these works unless the editor has indicated otherwise.

[Note: HTML markup is below. Please do not edit.]

Reviewers' comments:

Reviewer's Responses to Questions

**Comments to the Author**

1. Is the manuscript technically sound, and do the data support the conclusions?

The manuscript must describe a technically sound piece of scientific research with data that supports the conclusions. Experiments must have been conducted rigorously, with appropriate controls, replication, and sample sizes. The conclusions must be drawn appropriately based on the data presented.

Reviewer #1: Yes

Reviewer #2: Yes

Reviewer #3: Yes

-->

**********

-->

2. Has the statistical analysis been performed appropriately and rigorously?

Reviewer #1: Yes

Reviewer #2: Yes

Reviewer #3: Yes

-->

**********

-->

3. Have the authors made all data underlying the findings in their manuscript fully available?

The PLOS Data policy requires authors to make all data underlying the findings described in their manuscript fully available without restriction, with rare exception (please refer to the Data Availability Statement in the manuscript PDF file). The data should be provided as part of the manuscript or its supporting information, or deposited to a public repository. For example, in addition to summary statistics, the data points behind means, medians and variance measures should be available. If there are restrictions on publicly sharing data—e.g. participant privacy or use of data from a third party—those must be specified.

Reviewer #1: Yes

Reviewer #2: Yes

Reviewer #3: No

-->

**********

-->

4. Is the manuscript presented in an intelligible fashion and written in standard English?

PLOS ONE does not copyedit accepted manuscripts, so the language in submitted articles must be clear, correct, and unambiguous. Any typographical or grammatical errors should be corrected at revision, so please note any specific errors here.

Reviewer #1: Yes

Reviewer #2: Yes

Reviewer #3: Yes

-->

**********

-->

5. Review Comments to the Author

Please use the space provided to explain your answers to the questions above. You may also include additional comments for the author, including concerns about dual publication, research ethics, or publication ethics. (Please upload your review as an attachment if it exceeds 20,000 characters)

Reviewer #1: In this article, the authors question the existence of a dopaminergic continuum whose opposite ends would be apathy and impulsivity. To this end, apathy and impulsivity were assessed using dedicated scales in a large group of young healthy subjects. Their scores were correlated with performance on a stop-signal reaction time (SSRT) task. The authors hypothesized that impulsivity would be associated with reaction times on stop trials, whereas apathy would be associated with reaction times on go trials. Bayesian methods were used to explore these relationships. The authors report a positive association between impulsivity and apathy, which contradicts the hypothesis of opposite ends of a continuum. Overall, the results do not confirm their initial hypotheses.

The manuscript is clearly written, and the study addresses an interesting and relevant question. Importantly, the authors report negative findings, which are valuable for the field and may help guide future research. However, several issues need to be addressed.

1°) The authors contrast impulsivity (a personality trait that is not necessarily pathological) with apathy (a lack of motivation typically associated with neurological and psychiatric disorders).

In addition, apathy was assessed using the Lille Apathy Rating Scale (LARS), which was originally developed to detect and measure the severity of apathy in clinical populations. Its use in a healthy sample may therefore require further justification, as it extends beyond its initial validation context. The Apathy Motivation Index (AMI) would have been an interesting alternative, as it was specifically developed to measure motivation levels in both healthy individuals and patients. The authors state that the AMI has not been validated in French; however, it appears that a French validation has been published (Corveleyn et al., Frontiers in Psychology, 2023).

Moreover, the UPPS-P scale assesses a stable personality trait without a specific reference period, whereas the LARS refers to behaviors experienced over the past month and therefore captures a more state-like dimension. The implications of combining trait and state measures should be more explicitly discussed.

2°) The authors’ repeated reference to apathy (a pathological state) throughout the article is problematic. They studied healthy young volunteers whose motivation levels may vary, but likely none—or only a few—would be considered clinically apathetic. Furthermore, providing a table summarizing the demographic characteristics of the sample, along with the total and subscale scores for both instruments, would greatly enhance transparency and interpretability. At present, only correlation coefficients are reported, which makes the results difficult to interpret.

3°) In the discussion, in the section entitled “Trait dimensions do not map onto stop-signal indices,” the authors state that they explored the association between trait dimensions and performance on the SSRT task. While this is accurate for impulsivity (as the UPPS-P assesses a general tendency to act impulsively), it is not the case for apathy. The LARS does not assess a stable trait but rather a current behavioral state that is not necessarily related to personality. Although overall motivation levels vary between individuals, the LARS does not measure this as a trait dimension. This issue should be explicitly discussed.

4°) Although the authors mention some limitations, they should include a dedicated paragraph providing a more systematic discussion. For example, the very high proportion of women in the study population may have biased the results, as a substantial body of literature indicates sex differences in impulse control, with impulsivity generally being more frequent in men than in women. Addressing this point would further contextualize the findings.

5°) A major issue with the selected task is the absence of any reward component, despite the fact that both impulsivity and motivation are strongly related to reward processing. Impulsivity includes, among other features, increased reward-seeking and risk-taking behaviors. Similarly, apathy reflects a reduction in goal-directed behavior, which is largely driven by reward sensitivity. The absence of reward contingencies may have reduced the likelihood of observing meaningful associations. This important limitation is not mentioned by the authors and should be discussed in depth, as it may account for the negative results.

6°) Several redundancies (e.g., repeated paragraphs in the Introduction and Discussion or in the Methods and Results sections) could be streamlined to improve conciseness of the manuscript.

Reviewer #2: This is a well-executed study addressing a genuine gap in the literature, with satisfactory methodological rigor. The null findings are reported responsibly with appropriate Bayesian quantification. The main revisions needed before acceptance are:

(1) Explicit prior specification for Bayesian analyses. The manuscript does not report which prior distributions were used in the Bayesian Kendall correlation analyses.

(2) A discussion of multiple comparisons in the exploratory section. The exploratory analyses involve multiple separate correlation tests, yet the paper does not acknowledge how this affects the interpretation of the single positive result found. The goRT-negative urgency finding should be clearly framed as hypothesis-generating, and readers should be left with a sense of its evidential status relative to the paper's pre-specified hypotheses.

(3) More prominent acknowledgment of the highly female-skewed sample as a generalizability constraint.

(4) Provision of descriptive stop-signal performance statistics. The results section does not report basic descriptive statistics for the stop-signal task, including mean SSRT, mean goRT, mean stop-signal delay, etc. These values are necessary for readers to verify that the task performed as intended or to compare with prior studies.

Reviewer #3: The study by Michel et al. investigates the relationship between impulsivity, apathy, and inhibitory control using a well-powered stop-signal task, combined with validated multidimensional measures of impulsivity (UPPS-P) and apathy (LARS), and analyzed within a Bayesian framework.

The study is carefully designed, adheres to current methodological recommendations, and provides a clear and rigorous test of theoretically motivated hypotheses, alongside transparent exploratory analyses. Importantly, the findings contribute to the literature by delineating the limits of task-based measures in dissociating impulsivity and apathy in healthy individuals. I found this manuscript to be well written, methodologically sound, and theoretically well grounded. I have no major concerns regarding the study design, analyses, or interpretation of the results.

My comments below are therefore limited to minor points, mainly aimed at improving clarity, transparency, and readability.

1) A large number of analyses are reported, including several exploratory ones. Although the results are carefully presented, the overall pattern may be difficult to grasp for the reader. I think it would be helpful if the authors provided a graphical summary of the main results, in the spirit of the hypothesis-driven schematic shown in Fig.1, to offer an integrated overview of the key findings.

2) The authors state that the stop-signal task was implemented in accordance with the consensus recommendations (Verbruggen et al., 2019). In this context, it would be important to also report the descriptive statistics specified in these guidelines. In particular, stop-signal studies are expected to report the probability of go omissions, the probability of choice errors on go trials, mean goRT, the probability of responding on stop trials, the average SSD (given the use of a tracking procedure), mean stop-signal RT, and the RT of go responses on unsuccessful stop trials.

Reporting these descriptive statistics, for instance in a Table, would improve transparency and facilitate comparison with previous studies.

3) Related to my previous point, although individual values are shown for the total LARS and UPPS-P scores (Fig. 2A) and for some subscales (Fig. 3B), it would be informative to also report the mean ± SD for all subscale scores.

-->

**********

-->

6. PLOS authors have the option to publish the peer review history of their article (what does this mean?). If published, this will include your full peer review and any attached files.

If you choose “no”, your identity will remain anonymous but your review may still be made public.

**Do you want your identity to be public for this peer review?**  For information about this choice, including consent withdrawal, please see our Privacy Policy.

Reviewer #1: No

Reviewer #2: No

Reviewer #3: **Yes:**  Cecilia Neige

-->

**********

-->

[NOTE: If reviewer comments were submitted as an attachment file, they will be attached to this email and accessible via the submission site. Please log into your account, locate the manuscript record, and check for the action link "View Attachments". If this link does not appear, there are no attachment files.]

To ensure your figures meet our technical requirements, please review our figure guidelines: https://journals.plos.org/plosone/s/figures

You may also use PLOS’s free figure tool, NAAS, to help you prepare publication quality figures: https://journals.plos.org/plosone/s/figures#loc-tools-for-figure-preparation.

NAAS will assess whether your figures meet our technical requirements by comparing each figure against our figure specifications.

-->

# Author comment: Author response to Decision Letter 1

**DOI:** 10.1371/journal.pone.0352351.r002

6 May 2026

Revision of PONE-D-26-02739 as invited by the Academic Editor, Yansong Li

Dear Dr. Li,

Thank you for the thoughtful and constructive feedback on our submission. We greatly appreciate the time and effort that you and the reviewers devoted to evaluating our paper. We have carefully revised the manuscript in response to all comments raised. In this cover letter, we also wish to note that we have added a co-author to the authorship, Djamila Bennabi, in recognition of her substantial contribution to the revision of the manuscript.

We also clarify the role of the funding source as follows: This work was funded by the European Research Council (ERC) under the European Union’s Horizon Europe programme (Starting Grant No. 101039226, awarded to Mathieu Servant). The funders had no role in study design, data collection and analysis, decision to publish, or preparation of the manuscript. We have also removed the unpublished UPPS-P manual from the reference list and amended the corresponding in-text citation in accordance with PLOS ONE guidelines. To address the concern regarding data availability, we confirm that the anonymized data underlying the reported analyses are publicly available via the OSF repository indicated in the Data Availability Statement.

In addition, we expanded the descriptive reporting to facilitate verification of task performance and comparison with prior studies, corrected the in-text figure citations, and revised the formatting and file naming to ensure compliance with PLOS ONE style requirements. More broadly, we clarified the rationale for using the LARS in a healthy sample, specified the prior distribution used in the Bayesian analyses, added descriptive tables and a dedicated limitations paragraph, and framed the exploratory findings more cautiously.

We are grateful for these comments, which helped us improve the clarity, transparency, and interpretability of the paper. Below, we provide a detailed, point-by-point response to each comment and indicate the corresponding changes made in the revised version. The reviewers’ comments are reproduced in black, and our responses are provided in blue.

Best regards,

Mathieu Servant & Matthieu Béreau

On behalf of our co-authors

Reviewer #1: In this article, the authors question the existence of a dopaminergic continuum whose opposite ends would be apathy and impulsivity. To this end, apathy and impulsivity were assessed using dedicated scales in a large group of young healthy subjects. Their scores were correlated with performance on a stop-signal reaction time (SSRT) task. The authors hypothesized that impulsivity would be associated with reaction times on stop trials, whereas apathy would be associated with reaction times on go trials. Bayesian methods were used to explore these relationships. The authors report a positive association between impulsivity and apathy, which contradicts the hypothesis of opposite ends of a continuum. Overall, the results do not confirm their initial hypotheses. The manuscript is clearly written, and the study addresses an interesting and relevant question. Importantly, the authors report negative findings, which are valuable for the field and may help guide future research. However, several issues need to be addressed.

1°) The authors contrast impulsivity (a personality trait that is not necessarily pathological) with apathy (a lack of motivation typically associated with neurological and psychiatric disorders).In addition, apathy was assessed using the Lille Apathy Rating Scale (LARS), which was originally developed to detect and measure the severity of apathy in clinical populations. Its use in a healthy sample may therefore require further justification, as it extends beyond its initial validation context. The Apathy Motivation Index (AMI) would have been an interesting alternative, as it was specifically developed to measure motivation levels in both healthy individuals and patients. The authors state that the AMI has not been validated in French; however, it appears that a French validation has been published (Corveleyn et al., Frontiers in Psychology, 2023). Moreover, the UPPS-P scale assesses a stable personality trait without a specific reference period, whereas the LARS refers to behaviors experienced over the past month and therefore captures a more state-like dimension. The implications of combining trait and state measures should be more explicitly discussed.

R1-1: We thank the reviewer for this important comment. We agree that our original framing did not sufficiently distinguish clinical apathy from dimensional apathy-related variation in healthy individuals. In the revised manuscript, we clarify that both impulsivity and apathy can be studied in healthy individuals as well as in clinical populations. (e.g., Petitet et al., 2021, 2022).

We also agree that the use of the LARS in a healthy student sample requires more explicit justification. The LARS was originally developed in a clinical context and validated in Parkinson’s disease, although the initial study also included healthy control participants. In the revised manuscript, we now state this more explicitly. We explain that we selected the LARS because, when the study was designed, it was one of the few multidimensional apathy measures available in French. In addition, pairing an interviewer-administered apathy measure with a self-report impulsivity measure reduced the risk that the observed apathy–impulsivity association was driven solely by shared self-report method variance.

We agree that the Apathy Motivation Index (AMI) is particularly attractive for studies conducted in healthy populations, and in the revised manuscript we have corrected our statement regarding its French validation. Importantly, recruitment for the present study began on 1 June 2022, that is, before publication of the French validation of the AMI. We now acknowledge this validation in the manuscript and note that the French AMI may be preferable for future studies in healthy populations, or could be used alongside the LARS to compare interviewer-based and self-report assessments of apathy-related variation.

Finally, we agree that the distinction between trait- and state-like measures should be discussed more explicitly. In the revised manuscript, we clarify that the UPPS-P primarily assesses relatively stable impulsivity-related dispositions, whereas the LARS refers to thoughts, emotions, and activities over the previous four weeks. We therefore acknowledge that our analyses do not compare two perfectly matched trait measures, but rather relate trait-like impulsivity dispositions to recent apathy-related variation, and we identify this asymmetry as a limitation. At the same time, in a healthy sample, LARS scores may still capture meaningful inter-individual differences in subclinical motivational tendencies. Importantly, the positive association observed here is consistent with prior findings in healthy populations obtained with different self-report instruments, including the AMI, suggesting that the co-occurrence of apathy- and impulsivity-related tendencies is not solely driven by the particular instrument used in the present study.

2°) The authors’ repeated reference to apathy (a pathological state) throughout the article is problematic. They studied healthy young volunteers whose motivation levels may vary, but likely none—or only a few—would be considered clinically apathetic. Furthermore, providing a table summarizing the demographic characteristics of the sample, along with the total and subscale scores for both instruments, would greatly enhance transparency and interpretability. At present, only correlation coefficients are reported, which makes the results difficult to interpret.

R1-2: We agree that our sample consisted of healthy young adults, and that our aim was not to assess clinical apathy. In the revised manuscript, we clarify that both impulsivity and apathy can be studied dimensionally in healthy individuals as well as in clinical populations. Accordingly, in the present study, the LARS was used to characterize inter-individual variation in apathy-related tendencies in a non-clinical sample, rather than to identify a clinically apathetic subgroup. This dimensional approach is consistent with previous studies examining apathy and impulsivity in healthy and general-population samples (e.g., Petitet et al., 2021, 2022).

We also agree that providing descriptive statistics improves the transparency and interpretability of the results. We have therefore added a table (Table 1) summarizing the demographic characteristics of the final sample, together with descriptive statistics for the total and dimension scores of the UPPS-P and LARS instruments.

3°) In the discussion, in the section entitled “Trait dimensions do not map onto stop-signal indices,” the authors state that they explored the association between trait dimensions and performance on the SSRT task. While this is accurate for impulsivity (as the UPPS-P assesses a general tendency to act impulsively), it is not the case for apathy. The LARS does not assess a stable trait but rather a current behavioral state that is not necessarily related to personality. Although overall motivation levels vary between individuals, the LARS does not measure this as a trait dimension. This issue should be explicitly discussed.

R1-3. See R1-1. We fully agree that the LARS does not assess a stable trait, and we have revised the wording throughout the manuscript accordingly.

4°) Although the authors mention some limitations, they should include a dedicated paragraph providing a more systematic discussion. For example, the very high proportion of women in the study population may have biased the results, as a substantial body of literature indicates sex differences in impulse control, with impulsivity generally being more frequent in men than in women. Addressing this point would further contextualize the findings.

R1-4. We thank the reviewer for this important comment. We agree that the strong female skew in our sample should be discussed more explicitly as a limitation. In the revised manuscript, we have added a dedicated limitations paragraph. We now note that the high proportion of women may limit the generalizability of the findings and may also have reduced variability in impulsivity-related traits. More broadly, this concern is relevant because the literature indicates that sex/gender can influence multidimensional impulsivity profiles, although these effects are not uniform across all impulsivity domains. We also note that, while we initially fitted Bayesian regression models including age and sex as covariates, these analyses had negligible impact on the main results. However, given the marked sex imbalance in the present sample, our study was not well suited to testing sex-specific effects or interactions, and this should be borne in mind when interpreting the findings.

5°) A major issue with the selected task is the absence of any reward component, despite the fact that both impulsivity and motivation are strongly related to reward processing. Impulsivity includes, among other features, increased reward-seeking and risk-taking behaviors. Similarly, apathy reflects a reduction in goal-directed behavior, which is largely driven by reward sensitivity. The absence of reward contingencies may have reduced the likelihood of observing meaningful associations. This important limitation is not mentioned by the authors and should be discussed in depth, as it may account for the negative results.

We agree that the absence of explicit reward contingencies in the stop-signal task is a meaningful limitation, especially given that both impulsivity and apathy are closely linked to motivational and reward-related processes. In the revised manuscript, we now discuss this point more explicitly in the Limitations section. More specifically, we note that the neutral, non-rewarded structure of the stop-signal task may have reduced the likelihood of observing questionnaire–task associations, particularly for apathy-related variation and for impulsivity dimensions that are most strongly expressed under emotionally or motivationally salient conditions. We also clarify that this issue may help explain the null findings, because prior work using a rewarded task context in healthy individuals reported dissociable behavioral signatures of impulsivity and apathy, including reward-related modulation of response vigor for apathy (Petitet et al., 2022). Accordingly, we now frame the present null results more cautiously as boundary conditions specific to a neutral stop-signal task, rather than as evidence against any possible relationship between these constructs and action control.

6°) Several redundancies (e.g., repeated paragraphs in the Introduction and Discussion or in the Methods and Results sections) could be streamlined to improve conciseness of the manuscript.

R1-6. We agree that several passages could be streamlined for conciseness. In the revised manuscript, we reduced residual redundancies, especially where procedural details were repeated across the Methods and Results sections and where the opening of the General Discussion partially repeated material already introduced in the Introduction. These edits were intended to improve readability and conciseness without changing the content or interpretation of the results.

Reviewer #2: This is a well-executed study addressing a genuine gap in the literature, with satisfactory methodological rigor. The null findings are reported responsibly with appropriate Bayesian quantification. The main revisions needed before acceptance are:

(1) Explicit prior specification for Bayesian analyses. The manuscript does not report which prior distributions were used in the Bayesian Kendall correlation analyses.

R2-1: We thank the reviewer for pointing out this omission. We agree that the prior distribution used in the Bayesian analyses should be stated explicitly. In the revised manuscript, we now specify in the Statistical analyses section that the Bayesian Kendall’s τ-b correlation analyses were conducted in JASP using the default stretched beta prior (width = 1). We have also added the relevant methodological reference for this choice, van Doorn et al. (2018, The American Statistician), which is now cited in the manuscript.

(2) A discussion of multiple comparisons in the exploratory section. The exploratory analyses involve multiple separate correlation tests, yet the paper does not acknowledge how this affects the interpretation of the single positive result found. The goRT-negative urgency finding should be clearly framed as hypothesis-generating, and readers should be left with a sense of its evidential status relative to the paper's pre-specified hypotheses.

R2-2. We agree that the evidential status of the single positive result from the exploratory analyses should be stated more clearly. In the revised manuscript, we now explicitly note that these analyses were exploratory, involved multiple comparisons, and were not part of our pre-specified hypotheses. Accordingly, the positive association between mean goRT and negative urgency is framed as hypothesis-generating and requiring independent replication, rather than as a confirmatory result. More broadly, the manuscript now makes clearer that this exploratory finding should be interpreted differently from the primary hypothesis tests, for which the data provided moderate evidence against the predicted associations.

(3) More prominent acknowledgment of the highly female-skewed sample as a generalizability constraint.

R2-3. See R1-4.

(4) Provision of descriptive stop-signal performance statistics. The results section does not report basic descriptive statistics for the stop-signal task, including mean SSRT, mean goRT, mean stop-signal delay, etc. These values are necessary for readers to verify that the task performed as intended or to compare with prior studies.

R2-4. We agree that descriptive statistics for stop-signal performance should be reported to allow readers to evaluate whether the task behaved as intended and to facilitate comparison with prior studies. In the revised manuscript, we have therefore added a new table (Table 2) reporting the m

# Editor report: Decision Letter 1

**DOI:** 10.1371/journal.pone.0352351.r003

**Authors:** Yansong Li, Yansong Li

9 Jun 2026

Revisiting the relationship between impulsivity, apathy, and action control: Bayesian inference from a stop-signal task study

PONE-D-26-02739R1

Dear Dr. Matthieu Béreau,

We’re pleased to inform you that your manuscript has been judged scientifically suitable for publication and will be formally accepted for publication once it meets all outstanding technical requirements.

Within one week, you’ll receive an e-mail detailing the required amendments. When these have been addressed, you’ll receive a formal acceptance letter and your manuscript will be scheduled for publication.

An invoice will be generated when your article is formally accepted. Please note, if your institution has a publishing partnership with PLOS and your article meets the relevant criteria, all or part of your publication costs will be covered. Please make sure your user information is up-to-date by logging into Editorial Manager at Editorial Manager® and clicking the ‘Update My Information' link at the top of the page. For questions related to billing, please contact billing support.

If your institution or institutions have a press office, please notify them about your upcoming paper to help maximize its impact. If they’ll be preparing press materials, please inform our press team as soon as possible -- no later than 48 hours after receiving the formal acceptance. Your manuscript will remain under strict press embargo until 2 pm Eastern Time on the date of publication. For more information, please contact onepress@plos.org.

Kind regards,

Yansong Li

Academic Editor

PLOS One

Additional Editor Comments (optional):

Reviewers' comments:

# Editor report: Acceptance letter

**DOI:** 10.1371/journal.pone.0352351.r004

**Authors:** Yansong Li, Yansong Li

PONE-D-26-02739R1

PLOS One

Dear Dr. Servant,

I'm pleased to inform you that your manuscript has been deemed suitable for publication in PLOS One. Congratulations! Your manuscript is now being handed over to our production team.

At this stage, our production department will prepare your paper for publication. This includes ensuring the following:

* All references, tables, and figures are properly cited

* All relevant supporting information is included in the manuscript submission,

* There are no issues that prevent the paper from being properly typeset

You will receive further instructions from the production team, including instructions on how to review your proof when it is ready. Please keep in mind that we are working through a large volume of accepted articles, so please give us a few days to review your paper and let you know the next and final steps.

Lastly, if your institution or institutions have a press office, please let them know about your upcoming paper now to help maximize its impact. If they'll be preparing press materials, please inform our press team within the next 48 hours. Your manuscript will remain under strict press embargo until 2 pm Eastern Time on the date of publication. For more information, please contact onepress@plos.org.

You will receive an invoice from PLOS for your publication fee after your manuscript has reached the completed accept phase. If you receive an email requesting payment before acceptance or for any other service, this may be a phishing scheme. Learn how to identify phishing emails and protect your accounts at https://explore.plos.org/phishing.

If we can help with anything else, please email us at customercare@plos.org.

Thank you for submitting your work to PLOS ONE and supporting open access.

Kind regards,

PLOS ONE Editorial Office Staff

on behalf of

Dr. Yansong Li

Academic Editor

PLOS One
